# Gestational age dating using newborn metabolic screening: A validation study in Busia, Uganda

**DOI:** 10.7189/jogh.11.04012

**Published:** 2021-02-11

**Authors:** Scott P Oltman, Elizabeth A Jasper, Richard Kajubi, Teddy Ochieng, Abel Kakuru, Harriet Adrama, Martin Okitwi, Peter Olwoch, Moses Kamya, Bruce Bedell, Molly McCarthy, John Dagle, Prasanna Jagannathan, Tamara D Clark, Grant Dorsey, Larry Rand, Theodore Ruel, Elizabeth E Rogers, Kelli K Ryckman, Laura L Jelliffe-Pawlowski

**Affiliations:** 1 Department of Epidemiology and Biostatistics, University of California San Francisco School of Medicine, San Francisco, California, USA; 2 Preterm Birth Initiative, University of California San Francisco, San Francisco, California, USA; 3 Department of Epidemiology, University of Iowa College of Public Health, Iowa City, Iowa, USA; 4 Infectious Diseases Research Collaboration, Kampala, Uganda; 5 Department of Medicine, Makerere University College of Health Sciences, Kampala, Uganda; 6 Department of Pediatrics, University of Iowa, Iowa City, Iowa, USA; 7 Department of Medicine, Stanford University Medical Center, Stanford, California, USA; 8 Department of Medicine, University of California San Francisco School of Medicine, San Francisco, California, USA; 9 Department of Obstetrics, Gynecology, & Reproductive Sciences, University of California San Francisco, San Francisco, California, USA; 10 Department of Pediatrics, University of California San Francisco, San Francisco, California, USA

**Background** Limited ultrasound capacity in low-resource settings makes correct gestational age (GA) dating difficult. Previous work demonstrated that newborn metabolic profiles can accurately determine gestational age, but this relationship has not been evaluated in low-income countries. The objective of this study was to validate and adapt a metabolic GA dating model developed using newborn blood spots for use in a low-resource setting in rural Uganda.

**Methods** A cohort of pregnant women was followed prospectively and heel stick blood spots were collected from 666 newborns in Busia, Uganda at the time of delivery. They were dried, frozen, and shipped to the US where they were tested for 47 metabolites. Metabolic model performance was assessed using early ultrasound determined GA as the standard. Models tested included previously built multivariable models and models specifically adapted to the Busia population.

**Results** The previously built model successfully dated 81.2% of newborns within two weeks of their ultrasound GA. Only 4.8% of GAs were off by greater than three weeks. In the model adapted to the local population, 89.2% of GAs matched their corresponding ultrasound to within two weeks. The model-derived preterm birth rate was 7.2% compared to 5.9% by ultrasound.

**Conclusions** These results suggest that metabolic dating is a reliable method to determine GA in a low-income setting. Metabolic dating offers the potential to better elucidate preterm birth rates in low-resource settings, which is important for assessing population-level patterns, tailoring clinical care, and understanding the developmental trajectories of preterm infants.

In 2015, an estimated 1.055 million children under the age of five died from complications of preterm birth making it the leading cause of under-5 mortality worldwide ([1]). For the over 14 million preterm infants born annually [2] who do survive, complications including infection, neurological damage, sepsis, respiratory distress, necrotizing enterocolitis, and hearing and vision difficulties are common [3-8]. Moreover, a disproportionate share of preterm births occur in low-income countries. Specifically, the countries within Sub-Saharan Africa are estimated to account for 25% of global livebirths but 28.2% of global preterm births [2]. Particularly challenging, is that data from low-income countries is vulnerable to a high degree of uncertainty due to incomplete or lack of robust birth surveillance systems, absence of standardized definitions of preterm birth and viability, and lack of ability to accurately ascertain gestational age [2,9].

The inability to accurately ascertain gestational ages is a barrier to not only describing the epidemiology of preterm birth, but also to designing, implementing, and monitoring interventions that aim to improve health outcomes in this vulnerable population. Without reliable gestational age information, health systems can fail to efficiently utilize limited resources, and clinicians may miss opportunities for effective therapeutic interventions targeting issues specific to preterm infants. The current standard of care to determine gestational age is ultrasound dating early on in pregnancy, but ultrasound technology isn’t widely available in low-income countries. Alternative methods include using last menstrual period (LMP), clinical assessments like the new Ballard score [10] and Dubowitz score [11], and birthweight alone but none of these methods have been determined to yield an accurate gestational age – particularly when there is co-morbid growth restriction [9,12-15]. LMP has been shown to lack accuracy due to irregular menstrual cycles and unreliable recall, which is compounded in low-income countries by late presentation to antenatal care [9,13,15,16]. Clinical assessments like those included in the Ballard and Dubowitz scores are often imprecise due to skill and training discrepancies between evaluators [9,12,14,15], and difficulty when gauging the gestational age of growth-restricted newborns [9,12].

Recently, the World Health Organization (WHO) prioritized the development and validation of more reliable methods of gestational age dating in order to more accurately estimate preterm birth [17]. One such method pioneered by our group and others is to use metabolic markers from newborn heel-stick blood spots collected during routine newborn screening to determine gestational age at birth [18-20]. Our group and others have found that metabolites measured in newborn heel-stick samples can date ≥90% of newborns within two weeks of dating by early ultrasound – including in babies with intrauterine growth restriction (IUGR) [18,19].

The goal of this study was to validate and adapt a metabolic gestational age dating model [19] developed using newborn heel stick blood spots for use in a low-resource setting in rural Uganda. Additionally, we sought to compare the performance of the heel-stick model to a gestational age dating by metabolic profile using a cord blood sample collected within three hours of the heel stick sample. The successful development of novel methods to determine gestational age at birth could be crucial to more accurate assessments of preterm birth rates in low-income settings and has the potential to inform clinical care of infants born prematurely in these settings.

## METHODS

This prospective cohort study was nested within a double-blind, randomized clinical trial comparing the efficacy and safety of sulfadoxine-pyrimethamine (SP) vs dihydroartemisinin-piperaquine (DP) as intermittent preventative treatment of malaria during pregnancy [21]. The study took place between September 2016 and December 2017 in the Busia District of southeastern Uganda, which is characterized by intense malaria transmission. Individuals eligible for the study included women who were at least 16 years of age with a viable pregnancy between 12 and 20 weeks gestation determined by ultrasound, and who were HIV-uninfected. Written informed consent covering mothers and prospective infants was required from each participant along with agreements to: avoid taking medications that were outside of the study protocol, willingness to deliver at the hospital in Busia, and come to the clinic for any illness during pregnancy including a febrile event. Women were excluded if they had a history of antimalarial therapy during the current pregnancy, had known adverse responses to SP or DP, were in early or active labor, or had a currently active or chronic medical condition requiring inpatient evaluation. At enrollment, women underwent an initial standardized routine medical examination, including pre-natal ultrasound for gestational age dating, and were given a long-lasting insecticidal bed net. Participants were then randomized to receive either monthly SP or DP during pregnancy. Routine visits were conducted every four weeks at the clinic in Busia and any additional medical care was also received at the clinic, which was open every day. Additional information concerning study randomization and drug administration has been published previously [21].

The majority of women delivered their babies at the hospital adjacent to the clinic. Women who delivered at home were seen by study staff at the time of delivery or as soon as possible after delivery. At the time of delivery, a standardized assessment including information on congenital conditions, specimens collected, birthweight, infant sex, mode of delivery, and complications (pre-eclampsia, eclampsia, placental abruption, uterine rupture, fetal injury, cephalopelvic disproportion, and maternal hemorrhage) was completed. Preterm birth was defined as being born at less than 37 completed weeks of gestation.

Specimens collected for this study included umbilical cord blood and blood from newborn infant heel sticks. Cord blood was collected using a syringe inserted into the umbilical vein. From the syringe, 4-5 blood spots were collected. Newborn heel stick blood spots were obtained using standardized methodology from routine newborn screening [22]. Cord blood was collected at the time of delivery and blood from heel sticks within 3 hours of delivery in the majority of cases. In the occurrence of a home delivery, heel-stick blood spots were collected as soon as possible but cord blood was unable to be collected. Both specimen types were collected onto Whatman 903 protein saver filter paper cards. After allowing blood spots to dry, the filter paper cards were individually sealed in bags with desiccant pouches and stored in a liquid nitrogen-charged cryotank until shipped. Specimens were shipped approximately every two weeks from Kampala, Uganda to the University of California San Francisco using a liquid nitrogen-charged cryoshipper. Upon arrival in California, samples were removed, placed on dry ice, and sent to the State Hygienic Laboratory (SHL) in Ankeny, Iowa. Throughout the process, the cold-chain was maintained at -20°C or lower in order to prevent the degradation of acylcarnitines and amino acids. Metabolic markers measured by SHL included 2 enzymes, 1 hormone, 12 amino acids, and 32 acylcarnitines and were measured using either tandem mass spectrometry, time-resolved fluoroimmunoassay, or semiquantitative enzymatic assay. In depth methodology of these techniques as performed by SHL has been described previously [23,24].

Methods and protocols for the study were approved by the ethics committees of Makerere University School of Biomedical Sciences (Kampala, Uganda), the Uganda National Council for Science and Technology (Kampala, Uganda), and the Committee of Human Research at the University of California San Francisco.

### Statistical analyses

A natural log transformation was performed on all metabolites to reduce skewness and minimize the influence of outliers. Given that our sample size was too small to create a reliable small for gestational age measurement, two independent alternative methods were used to determine the 10th percentile birthweight cutoffs by gestational age and sex. The first method utilized the Intergrowth-21st international standardized growth curves [25,26]. The second method used a WHO calculator [27] that creates standardized growth curves based on the mean birthweight of infants born at 40 weeks gestation in the study population (3178.4g). Continuous variables described using mean and standard deviation (SD) and categorical variables using frequencies and proportions. Univariable analyses were performed on metabolites and clinical characteristics comparing term vs preterm infants using *t* tests and χ^2^ or Fischer exact tests (if n ≤5 within a category) for continuous and categorical variables, respectively.

Models using metabolic data to predict ultrasound dated prematurity were evaluated in several steps. First, the Ryckman model developed previously to predict gestational ages of an infant cohort born Iowa was evaluated (validated) for its ability to predict ultrasound-based gestational age in this cohort (Table S1 in the **Online Supplementary Document**) [19]. Second, a model was built specifically from the Busia data using cross-validated stepwise multivariable logistic regression. Metabolites with approximately normal distributions (skew<|1.0|), birthweight, and sex were permitted to enter the model and a *P* < 0.05 was required to remain in the model. Overall model performance was evaluated using area under receiver operating characteristic curve (AUC) and odds ratios (OR) with 95% confidence intervals (CI) for individual variables. The variables that formed the final model were then input into a linear regression to obtain specific predicted gestational ages for each infant. Performance of the linear model was examined using adjusted R^2^. Correspondence of the model-determined gestational ages to the ultrasound-determined gestational ages was also examined specifically in infants born SGA. The ability of both models to correctly classify preterm and term birth was assessed using sensitivity, specificity, positive predictive value (PPV), negative predictive value (NPV), and accuracy. This same model building and evaluating process and the same comparative analyses were performed using the results from the cord blood specimens with overall results compared to those from the heel-stick samples.

All analyses were performed using SAS 9.4 (SAS institute, Cary, NC, USA).

## RESULTS

Heel-stick blood spots were collected from 666 of the 687 (96.9%) live born infants in the trial, of those, 39 (5.9%) had been born preterm per ultrasound dating. SGA rates in this population were 17% and 13.5% by intergrowth and WHO respectively. Infants born preterm were more likely than their term counterparts to have lower birthweights, and infants born term were more likely to be singletons than those born preterm. We found no statistically significant difference in sex, SGA, or age at specimen collection between infants born term and preterm (**Table 1**). There were 640 infants with blood spots from cord blood, and 36 (5.6%) were born preterm as determined by ultrasound. Differences between term and preterm infants within this subset were similar to those above (Table S2 in the **Online Supplementary Document**).

**Table 1:** Univariable analyses of clinical characteristics in infants born term and preterm with heel-stick blood spots collected

	Heel			
	**Total**	**Term**	**Preterm**	***P-*value**
	**n = 666**	**n = 627**	**n = 39**	
Gestational age* (weeks), mean (SD)	39.1 (1.7)	39.4 (1.2)	34.4 (2.0)	<0.001
Gestational age category* (completed weeks), n (%):				<0.001
≥37	627 (94.1)	627 (100)	0 (0.0)	
32-36	36 (5.4)	0 (0.0)	36 (92.3)	
<32	3 (0.5)	0 (0.0)	3 (7.7)	
Birthweight (grams), mean (SD)	3036.8 (455.6)	3086.7 (401.7)	2235.1 (523.5)	<0.001
Birthweight category (grams), n (%):				<0.001
≥4000	12 (1.8)	12 (1.9)	0 (0)	
3500-3999	96 (14.4)	96 (15.3)	0 (0)	
3000-3499	263 (34.5)	261 (41.6)	2 (5.1)	
2500-2999	237 (35.6)	225 (35.9)	12 (30.8)	
2000-2499	42 (6.3)	30 (4.8)	12 (30.8)	
1500-1999	13 (2.0)	3 (0.5)	10 (25.6)	
1000-1499	3 (0.5)	0 (0.0)	3 (7.69)	
<1000	0 (0.0)	0 (0.0)	0 (0.0)	
Age at collection (hours), mean (SD)	1.78 (4.22)	1.7 (4.3)	2.4 (3.5)	0.366
Sex n (%):				0.705
Male	327 (49.1)	309 (49.3)	18 (46.2)	
Female	339 (50.9)	318 (50.7)	21 (53.9)	
Multiple gestation	25 (3.8)	17 (2.7)	8 (20.5)	<0.001
SGA n (%):				
Intergrowth	113 (17.0)	108 (17.2)	5 (12.8)	0.477
Busia specific	90 (13.5)	81 (12.9)	9 (23.1)	0.072
Treatment arm				0.034†
DP	332 (49.9)	319 (50.9)	13 (33.3)	
SP	334 (50.1)	308 (49.1)	26 (66.7)	

SD – standard deviation, SGA – small for gestational age, DP – dihydroartemisinin-piperaquine, SP – sulfadoxine-pyrimethamine

*As measured by ultrasound between 12-20 weeks.

†Each infant counted even if part of a multiple gestation (insignificant when calculated by maternal delivery). Continuous variables described using mean and standard deviation and categorical variables using frequencies and proportions. *t* tests and χ^2^ tests for continuous and categorical variables respectively were used to compare cases and control.

Metabolic analyses included 47 routinely measured newborn screening metabolites. Of these metabolites, 13 were excluded for skewness>|1.0|, leaving 34 metabolites for univariable analysis. There were 18 metabolites (13 acylcarnitines, 4 amino acids, and TSH) that differed significantly between term and preterm infants. Those most strongly associated (*P* value <0.001) with preterm birth in univariable analysis were C4, C4-DC, C4-OH, C5, C8, phenylalanine, tyrosine, and TSH (**Table 2**). Among cord blood measurements, 12 metabolites differed significantly between term and preterm infants (Table S3 in the **Online Supplementary Document**).

**Table 2:** Univariable analyses of mean levels of metabolic makers in infants born term and preterm from heel-stick blood spots*

	Heel			
	**Term (n = 627)**		**Preterm (n = 39)**	
**Variable**	**Mean (SD)**	**95% CI**	**Mean (SD)**	**95% CI**
**Acylcarnitines:**				
Free Carnitine	3.02 (0.34) †	2.99 to 3.05	3.13 (0.28) †	3.04 to 3.22
C2	3.08 (0.35)	3.05 to 3.11	3.15 (0.35)	3.03 to 3.26
C3	0.11 (0.39) †	0.08 to 0.14	0.25 (0.43) †	0.11 to 0.39
C4	-1.6 (0.35) ‡	-1.62 to -1.57	-1.31 (0.37) ‡	-1.44 to -1.19
C4-DC	-1.79 (0.39) ‡	-1.82 to -1.76	-2.08 (0.31) ‡	-2.18 to -1.98
C4-OH	-2.82 (0.38) ‡	-2.85 to -2.79	-2.56 (0.49) ‡	-2.72 to -2.4
C5	-2.1 (0.38) ‡	-2.13 to -2.07	-1.72 (0.44) ‡	-1.87 to -1.58
C5-OH	-2.34 (0.34)	-2.37 to -2.31	-2.3 (0.35)	-2.41 to -2.19
C6	-3.15 (0.35)	-3.18 to -3.12	-3.05 (0.39)	-3.17 to -2.92
C8	-3.5 (0.45) ‡	-3.54 to -3.46	-3.23 (0.52) ‡	-3.4 to -3.06
C10	-3.26 (0.51) †	-3.3 to -3.22	-3.05 (0.63) †	-3.25 to -2.85
C12	-2.18 (0.69)	-2.23 to -2.13	-2.21 (0.62)	-2.41 to -2.01
C12:1	-3.3 (0.58) †	-3.35 to -3.26	-3.02 (0.82) †	-3.29 to -2.76
C14	-1.7 (0.42) †	-1.73 to -1.66	-1.55 (0.45) †	-1.7 to -1.4
C14:1	-2.59 (0.6) †	-2.63 to -2.54	-2.37 (0.68) †	-2.59 to -2.15
C16	0.97 (0.35)	0.95 to 1	0.97 (0.33)	0.87 to 1.08
C16:1	-2.06 (0.38) †	-2.09 to -2.03	-1.86 (0.46) †	-2.01 to -1.71
C16:1-OH	-2.95 (0.34) †	-2.97 to -2.92	-3.11 (0.33) †	-3.22 to -3.01
C18	0.03 (0.36)	0 to 0.06	-0.03 (0.3)	-0.13 to 0.07
C18:1	-0.09 (0.34)	-0.11 to -0.06	0.01 (0.35)	-0.1 to 0.12
C18:2	-1.76 (0.38)	-1.79 to -1.73	-1.84 (0.29)	-1.94 to -1.75
**Amino acids/intermediates:**				
Alanine	5.53 (0.28) †	5.51 to 5.56	5.42 (0.32) †	5.32 to 5.53
Arginine	2.12 (0.51)	2.08 to 2.16	2.14 (0.49)	1.98 to 2.3
Citrulline	2.45 (0.23)	2.43 to 2.47	2.47 (0.38)	2.35 to 2.59
Glutamate	5.18 (0.26)	5.16 to 5.2	5.17 (0.24)	5.09 to 5.25
Leucine	4.7 (0.22) †	4.68 to 4.72	4.8 (0.25) †	4.72 to 4.89
Methionine	3.04 (0.21)	3.03 to 3.06	3.01 (0.28)	2.92 to 3.11
Ornithine	3.21 (0.24)	3.19 to 3.23	3.14 (0.31)	3.04 to 3.24
Phenylalanine	4.2 (0.21) ‡	4.18 to 4.21	4.34 (0.35) ‡	4.22 to 4.45
Succinylacetone	-0.54 (0.17)	-0.55 to -0.53	-0.52 (0.2)	-0.59 to -0.46
Tyrosine	3.9 (0.24) ‡	3.88 to 3.92	4.12 (0.47) ‡	3.97 to 4.27
Valine	4.66 (0.2)	4.65 to 4.68	4.67 (0.2)	4.6 to 4.73
**Hormones:**				
17-hydroxyprogesterone	3.19 (0.63)	3.14 to 3.24	3.21 (0.74)	2.97 to 3.44
Thyroid stimulating hormone	3.33 (0.71) ‡	3.28 to 3.39	2.81 (0.57) ‡	2.62 to 3

CI – confidence interval, SD – standard deviation

*All variables are natural log transformed.

†*P* < 0.05.

‡*P* < 0.001.

The Ryckman model (Table S1 in the **Online Supplementary Document**) identified 48 (7.2%) infants as being born preterm. Using birthweight alone identified 128 (19.2%) as being born preterm. The Ryckman model gestational ages matched ultrasound gestational ages to within two weeks in 81.2% of infants, and only 4.8% were off by more than three weeks (**Table 3**). Additionally, the Ryckman model accurately classified 95% of infants as term or preterm with a sensitivity of 69.2% and a specificity of 96.7% (**Table 4**). When looking at SGA specifically, the Ryckman model underestimated gestational age by an average of 2.04 and 2.09 weeks, and gestational ages matched ultrasound gestational ages within two weeks for 43.4% and 36.6% of infants when using intergrowth and WHO SGA, respectively (**Table 5**).

**Table 3:** Weeks of difference between heel-stick model determined gestational ages and ultrasound determined gestational ages

	Ryckman heel		Busia heel	
	**Frequency (%)**	**Cumulative frequency (%)**	**Frequency (%)**	**Cumulative frequency (%)**
Perfect match*	22 (3.3)	22 (3.3)	43 (6.5)	43 (6.5)
≤1 week	283 (42.5)	305 (45.8)	376 (56.5)	419 (62.9)
≤2 weeks	236 (35.4)	541 (81.2)	175 (26.3)	594 (89.2)
≤3 weeks	94 (14.1)	635 (95.4)	56 (8.4)	650 (97.6)
≤4 weeks	15 (2.3)	650 (97.6)	12 (1.8)	662 (99.4)
≤5 weeks	11 (1.7)	661 (99.3)	3 (0.5)	665 (99.9)
5+ weeks	5 (0.8)	666 (100.0)	1 (0.2)	666 (100.0)

*Perfect match is ±1/2 d.

**Table 4:** Classification statistics of the heel-stick models used to determine preterm birth

	Heel	
	**Ryckman**	**Busia**
Sensitivity	69.2	61.5
Specificity	96.7	98.7
Positive predictive value	56.3	75.0
Negative predictive value	98.1	97.6
Accuracy	95.0	96.5

**Table 5:** Weeks of difference between heel-stick model determined gestational ages and ultrasound determined gestational ages in infants born SGA*

	Ryckman heel				Busia heel			
	**Intergrowth SGA**		**Busia specific SGA**		**Intergrowth SGA**		**Busia Specific SGA**	
	**No SGA (n = 553)**	**SGA (n = 113)**	**No SGA (n = 576)**	**SGA (n = 90)**	**No SGA (n = 553)**	**SGA (n = 113)**	**No SGA (n = 576)**	**SGA (n = 90)**
Perfect match†	22 (4.0)	0 (0.0)	22 (3.8)	0 (0.0)	35 (6.3)	8 (7.1)	38 (6.6)	5 (5.6)
0-1 week	269 (48.6)	14 (12.4)	272 (47.2)	11 (12.2)	334 (60.4)	42 (37.2)	349 (60.6)	27 (30.0)
1-2 weeks	201 (36.4)	35 (31.0)	214 (37.2)	22 (24.4)	140 (25.3)	35 (31.0)	146 (25.4)	29 (32.2)
2-3 weeks	52 (9.4)	42 (37.2)	58 (10.1)	36 (40.0)	32 (5.8)	24 (21.2)	32 (5.6)	24 (26.7)
3-4 weeks	4 (0.7)	11 (9.7)	4 (0.7)	11 (12.2)	9 (1.6)	3 (2.7)	8 (1.4)	4 (4.4)
4-5 weeks	3 (0.5)	8 (7.1)	4 (0.7)	7 (7.8)	3 (0.5)	0 (0.0)	3 (0.5)	0 (0.0)
5+ weeks	2 (0.4)	3 (2.7)	2 (0.4)	3 (3.3)	0 (0.0)	1 (0.9)	0 (0.0)	1 (1.1)

SGA – small for gestational age

*Values are frequency (column %). Busia specific SGA determined using WHO calculator.

†Perfect Match is ±1/2 d.

The Busia specific multivariable model showed the strongest performance identifying preterm infants (AUC = 0.953 95% CI = 0.921-0.985) and was comprised of 8 variables including birthweight, TSH, alanine, tyrosine, C4-DC, C5, C10, and C16:1-OH (**Table 6**). This model identified 30 (4.5%) preterm infants, and when used to classify infants as preterm or term, this model had a sensitivity of 61.5% and a positive predictive value of 75.0% as compared to the Ryckman model’s values of 69.2% and 56.3%, respectively. Other classification metrics were quite similar to the Ryckman model (**Table 4**). The Busia model was able to match ultrasound gestational ages within two weeks for 89.2% of newborns, and only 2.5% were off by more than three weeks (**Table 3**). It was also more robust to SGA, matching ultrasound within two weeks for 75.3% and 67.8% of infants but still underestimating gestational age by an average of 1.05 and 1.08 weeks when using intergrowth and WHO SGA, respectively (**Table 5**).

**Table 6:** Cross validated multivariable logistic heel-stick model built within the Busia cohort

Heel		
**AUC = 0.953 95% CI = 0.921-0.985**		
**Variable**	**Parameter estimate**	**OR (95% CI)**
Intercept	8.63	NA
Birthweight (per 100g)	-0.55	0.58 (0.48-0.69)
Thyroid stimulating hormone	-0.72	0.49 (0.25-0.96)
Alanine	-2.53	0.08 (0.01-0.49)
Tyrosine	-0.94	0.39 (0.13-1.13)
C4-DC	-2.13	0.12 (0.02-0.71)
C5	-1.95	0.14 (0.03-0.6)
C10	2.84	17.2 (3.73-79.27)
C16:1-OH	2.96	19.25 (2.51-147.72)

AUC – area under curve, OR – odds ratio, CI – confidence interval

The multivariable model built using cord blood displayed good performance (AUC = 0.935 95% CI = 0.894-0.977) and consisted of 6 variables: birthweight, alanine, C4, C4-DC, C4-OH, and C16:1-OH (Table S4 in the **Online Supplementary Document**). The cord blood model had lower sensitivity (52.8%) than the heel blood models, but otherwise, was comparable (Table S5 in the **Online Supplementary Document**). The cord blood model’s gestational ages matched ultrasound gestational ages in very similar proportions as the heel model for both the entire cohort and the SGA infants (Table S6 and S7 in the **Online Supplementary Document**).

## DISCUSSION

Gathering reliable epidemiologic information concerning preterm birth in low-income countries is crucial in order to better understand the burden of morbidity and mortality stemming from being born preterm and in order to allocate resources to alleviate preterm birth [2,17]. Currently, our best methods to determine gestational age in the absence of an early antenatal care ultrasound is to depend on LMP, clinical assessments at birth, or in some cases, birthweight alone all of which can be unreliable [9,12-15]. Our study used newborn screening-based metabolic models to estimate gestational age at birth, and generated preterm birth rates similar to that based on ultrasound (Ryckman model = 7.2%, Busia model = 4.5%, Ultrasound = 5.9%). Moreover, the vast majority of calculated gestational ages were within two weeks of ultrasound determined gestational age (Ryckman model = 81.2%, Busia model = 89.2%). In comparison to LMP and neonatal assessments like the Ballard and Dubowitz scores, our models of estimation perform similarly to those methods used in ideal settings. If, however, there is late entry to antenatal care for LMP or a lack of time and resources to devote to training and performing neonatal assessments (both common in low resource settings), then the accuracy of these alternative methods drastically declines (>±3 weeks) whereas our model should remain robust [9,12-15]. Overall, our results suggest that heel stick-based metabolic modeling could be a more scalable and accurate way to measure gestational age compared to traditional physical exam-based scores in the absence of ultrasound.

The Ryckman model was originally developed within a cohort of infants born in Iowa, and was one of three models built by research groups working cooperatively in North America [18-20]. Despite the population in Iowa being remarkably different from our validation population in Busia, the model still performed reasonably well suggesting it may be generalizable. Nevertheless, we built a model exclusively within the Busia population to elucidate any unique metabolic signals between populations. We found that all seven metabolic variables of importance to the Busia model were also present in the Ryckman model suggesting that metabolic profiles may only need to be calibrated to specific populations rather than completely altered.

Of particular importance within low-income countries where rates of infants born SGA has been estimated to reach 27% [28], is the impact of growth restriction on the ability to determine gestational age. Given the unique risks for mortality and morbidity associated with SGA [29], it is important for a measure of gestational age to differentiate between an infant born preterm, SGA, or both. Clinical assessments including those by Ballard and Dubowitz tend to be prone to the underestimation of gestational age in infants born SGA as they are more likely to exhibit less mature physical characteristics and behavior [9], and using birthweight alone will also underestimate gestational age. In our study, the Ryckman model underestimated gestational age in infants born SGA by an average of about 2 weeks, but the Busia model reduced this to an average underestimation of a little over 1 week. While underestimation remains a problem, our results suggest that tuning models to the local population may help in the discrimination of infants born SGA.

The models from heel-stick blood spots and cord blood were compared to determine if both methods could be used interchangeably. Having multiple methodologies that are reliable would prove useful in situations where heel-sticks aren’t ideal – cord blood is readily available – or where cultural customs make one collection type preferential. We found, however, that models built from heel-stick blood spots are better by most metrics including correspondence to ultrasound gestational ages, classification measures, and performance within SGA infants. This boosted performance is likely the result of the heel-stick models relying solely on samples taken directly from the infant, which may be more representative of the infant’s physiology as compared to cord specimens.

The estimated preterm birth rate in Sub-Saharan Africa is approximately 12.0% (CI 8.6%-16.7%) though it varies greatly between countries and studies based on a number of factors including access to health care, gestational age measurement tool, and differing definitions of viability and preterm birth [2]. In our study, both the ultrasound determined preterm birth rate and model determined rates were much lower than would be expected for the region. One possible explanation for this discrepancy is that the women in our trial were healthier and had better access to prenatal care than general population. Women enrolled in the clinical trial received extra antenatal care visits, antibiotics and other medications, prenatal supplements, and malaria chemoprevention that a majority of women in low-income regions have limited access to. It is also possible that the difference reflects some overestimation in the global estimates, since they often include birthweight as surrogate for prematurity.

### Strengths and limitations

This study has important strengths and limitations to consider. A major strength of the study is the ability to compare our model determined gestational ages to the gold standard early pregnancy ultrasounds, which are relatively rare in low-income countries. Additionally, our study was facilitated by a research infrastructure that could acquire blood spots and maintain dry cold-chain throughout storage and shipping making the mass spectrometry analyses more reliable as we know metabolite concentrations can be affected by a number of environmental factors [23]. Likewise, this necessary infrastructure is a significant obstacle to scale up this particular method of gestational age determination. Another potential shortcoming of our study is that despite participant selection being population based, the women in the study received differential treatment from the rest of the population possibly limiting the generalizability of our results. Finally, the Busia specific models relied on a small sample, which restricted the model to using fewer variables and constrained our ability to independently validate the model in a subset of the population.

While metabolic gestational age dating may provide an immediate impact by improving preterm birth epidemiology, there are current limitations to the utility of using metabolic profiles to inform treatment. Currently, with sufficient infrastructure, the turnaround time for newborn screening is within 1-2 days. To our knowledge, there is not currently an available facility equipped to handle high throughput mass spectrometry of newborn samples within Uganda or East Africa, which necessitated shipment of samples out of country resulting in significant delays. Future efforts should focus on capacity development in-region not only to expedite gestational age dating, but to facilitate potentially life-saving diagnoses that accompany newborn screening [24].

## CONCLUSIONS

Our findings support the notion that newborn screening metabolic profiles from heel-stick blood spots can reliably determine gestational age at birth with the additional utility of accurately estimating preterm birth rates. Utilizing this novel tool in a more widespread effort can improve preterm birth surveillance and epidemiology. In the future, if developed, metabolic profiles may help to inform treatment and clinical management shortly after delivery.

## Additional material

Online Supplementary Document
