# Supplementary material for: Gestational age dating using newborn metabolic screening: A validation study in Busia, Uganda
Source: J Glob Health. 2021 Feb 11;11:04012. doi: 10.7189/jogh.11.04012 (PMC7916447; doi:10.7189/jogh.11.04012)
Supplement: Online Supplementary Document [file jogh-11-04012-s001.pdf]

Supplemental Table S1. Final metabolite model in the model-building dataset (n = 153,342) for prediction of gestational age from *Ryckman et al.*

	Metabolite		Metabolite Squared		Metabolite Cubed	
Metabolite	Coefficient	SE	Coefficient	SE	Coefficient	SE
TSH	0.01	8.8×10^{-4}	-8.8×10^{-5}	1.2×10^{-5}	1.3×10^{-7}	2.3×10^{-8}
17-OHP	−0.05	5.2×10^{-4}	1.1×10^{-4}	3.5×10^{-6}	N/A	N/A
GALT	−0.07	0.01	3.5×10^{-3}	7.5×10^{-4}	N/A	N/A
ALA	9.6×10^{-3}	2.7×10^{-4}	-1.3×10^{-5}	6.3×10^{-7}	5.3×10^{-9}	3.7×10^{-10}
ARG	−0.02	1.5×10^{-3}	2.9×10^{-5}[a]	1.0×10^{-5}	N/A	N/A
LEU	−0.01	3.3×10^{-4}	1.7×10^{-5}	8.3×10^{-7}	-4.3×10^{-9}	2.1×10^{-10}
MET	0.03	1.2×10^{-3}	-2.3×10^{-4}	1.2×10^{-5}	3.5×10^{-7}	1.8×10^{-8}
PHE	−0.01	8.3×10^{-4}	4.2×10^{-5}	4.7×10^{-6}	-2.9×10^{-8}	3.9×10^{-9}
TYR	-5.3×10^{-3}	1.2×10^{-4}	N/A	N/A	N/A	N/A
VAL	0.02	6.0×10^{-4}	-3.6×10^{-5}	2.5×10^{-6}	2.0×10^{-8}	1.0×10^{-9}
C2	−0.10	4.4×10^{-3}	2.0×10^{-3}	1.1×10^{-4}	-1.2×10^{-5}	8.5×10^{-7}
C3	0.06	5.6×10^{-3}	N/A	N/A	N/A	N/A
C4	−0.20	0.03	N/A	N/A	N/A	N/A
C5	−9.29	0.26	−6.93	0.78	6.81	0.43
C5:1	−3.89	0.56	N/A	N/A	N/A	N/A
C5-OH	−0.36	0.08	N/A	N/A	N/A	N/A
C3-DC	0.91	0.1	N/A	N/A	N/A	N/A
C4-DC	18.56	0.41	−50.43	1.67	40.19	1.92
C5-DC	−25.06	0.92	85.79	8.6	−118.09	23.9
C6	10.16	0.31	−2.24	0.42	N/A	N/A
C8	−1.47	0.2	0.11	0.02	-2.0×10^{-3}	3.0×10^{-4}
C8:1	13.29	0.5	−52.96	2.51	55.61	3.64
C10	4.99	0.32	−10.87	0.91	3.97	0.52
C10:1	−2.33	0.27	N/A	N/A	N/A	N/A
C12	2.47	0.19	−4.00	0.37	2.1	0.22
C12:1	5.17	0.3	−13.00	0.85	9.71	0.72
C6-DC	−4.20	0.36	9.65	2.13	N/A	N/A

C14	−3.03	0.32	1.94	0.48	N/A	N/A
C16	2.99	0.07	−0.58	0.02	0.04	1.5×10^{-3}
C16:1	−9.73	0.55	18.06	1.63	−10.40	1.59
C18	−7.96	0.23	4.96	0.2	−1.02	0.05
C18:1	4.09	0.18	−1.70	0.11	0.24	0.02
C18:2	−4.22	0.19	3.01	0.35	−1.22	0.17
C14-OH	27.02	1.83	−287.34	35.03	N/A	N/A
C16-OH	10.91	2.58	−239.18	62.78	1585.56[a]	460.58
C16:1-OH	7.32	1.09	18.72[b]	8.87	N/A	N/A
C18:1-OH	3.84	0.82	N/A	N/A	N/A	N/A
Constant	36.72	0.11	N/A	N/A	N/A	N/A

GALT, galactose-1-phosphate uridyl transferase; N/A, not available; 17-OHP, 17-hydroxyprogesterone TSH, thyroid-stimulating hormone.

All terms are significant at $P < 0.001$ unless otherwise noted.

[a]$P<0.01$ [b]$0.01\leq P<0.05$.

Ryckman. Metabolic predictors of gestational age. Am J Obstet Gynecol 2016.

Supplemental Table S2. Univariable analyses of clinical characteristics in infants born term and preterm with cord blood spots collected.

	**Cord**			
	Total	Term	Preterm	P-value
	n = 641	n = 604	n = 36	
Gestational Age* (weeks) mean (SD)	39.2 (1.7)	39.4 (1.2)	34.4 (2.0)	<0.0001
Gestational Age Category* (completed weeks), n (%)				
≥37 weeks	604 (94.4)	604 (94.4)	0 (0.0)	<0.0001
32-36 weeks	33 (5.2)	0 (0.0)	33 (91.7)	
<32 weeks	3 (0.5)	0 (0.0)	3 (8.3)	
Birthweight (grams), mean (SD)	3045.1 (456.5)	3093.8 (402.7)	2229.7 (534.1)	<0.0001
Birthweight category (grams), n (%)				<0.0001
≥4000g	12 (1.9)	12 (2.0)	0 (0.0)	
3500-3999g	95 (14.8)	95 (15.7)	0 (0.0)	
3000-3499g	256 (40.0)	254 (42.1)	2 (5.6)	
2500-2999g	221 (34.5)	210 (34.8)	11 (30.6)	
2000-2499g	41 (6.4)	30 (5.0)	11 (30.6)	
1500-1999g	12 (1.9)	3 (0.5)	9 (25.0)	
1000-1499g	3 (0.5)	0 (0.0)	3 (8.3)	
<1000g	0 (0.0	0 (0.0)	0 (0.0)	
Age at Collection (hours), mean (SD)	NA	NA	NA	NA
Sex n (%)				0.5814
Male	313 (48.9)	297 (49.2)	16 (44.4)	
Female	327 (51.1)	307 (50.8)	20 (55.6)	
Multiple gestation	25 (3.9)	17 (2.8)	8 (22.2)	<0.0001
SGA n (%)				
Intergrowth	106 (16.6)	101 (16.7)	5 (13.9)	0.657
Busia specific	76 (12.6)	76 (12.6)	8 (22.2)	0.096
Treatment arm				0.0761**
DP	323 (50.5)	310 (51.3)	13 (36.1)	
SP	317 (49.5)	294 (48.7)	23 (63.9)	

*As measured by ultrasound between 12-20 weeks. **Each infant counted even if part of a multiple gestation. Continuous variables described using mean and standard deviation and categorical variables using frequencies and proportions. T-tests and Chi-squared tests for continuous and categorical variables respectively were used to compare cases and controls

Supplemental Table S3. Univariable analyses of mean levels of metabolic makers in infants born term and preterm from cord blood spots.

	**Cord**			
	Term (n = 627)		Preterm (n = 39)	
Variable	Mean (sd)	95% CI	Mean (sd)	95% CI
**Acylcarnitines**				
Free Carnitine	**2.84 (0.31)**	**2.81 to 2.86**	**2.99 (0.36)**	**2.87 to 3.11**
C-2	**2.86 (0.34)**	**2.83 to 2.88**	**2.98 (0.4)**	**2.84 to 3.11**
C-3	-0.14 (0.35)	-0.17 to -0.11	-0.11 (0.31)	-0.22 to -0.01
C-4	**-1.79 (0.32)**	**-1.81 to -1.76**	**-1.41 (0.55)**	**-1.6 to -1.23**
C-4DC	**-1.91 (0.39)**	**-1.94 to -1.88**	**-2.15 (0.4)**	**-2.28 to -2.01**
C-4OH	**-2.83 (0.43)**	**-2.86 to -2.79**	**-2.41 (0.69)**	**-2.64 to -2.17**
C-5	**-2.44 (0.39)**	**-2.47 to -2.41**	**-2.09 (0.51)**	**-2.26 to -1.92**
C-5OH	-2.59 (0.34)	-2.62 to -2.56	-2.56 (0.41)	-2.7 to -2.42
C-6	**-3.13 (0.32)**	**-3.16 to -3.11**	**-2.91 (0.44)**	**-3.06 to -2.76**
C-8	**-3.56 (0.38)**	**-3.59 to -3.53**	**-3.39 (0.35)**	**-3.51 to -3.27**
C-10	-3.4 (0.42)	-3.43 to -3.36	-3.31 (0.4)	-3.45 to -3.18
C-12	-2.61 (0.63)	-2.66 to -2.56	-2.7 (0.41)	-2.84 to -2.56
C-12:1	-3.59 (0.43)	-3.63 to -3.56	-3.52 (0.38)	-3.65 to -3.39
C-14	-2.08 (0.37)	-2.11 to -2.05	-2.02 (0.3)	-2.13 to -1.92
C-14:1	-2.97 (0.52)	-3.02 to -2.93	-2.89 (0.38)	-3.01 to -2.76
C-16	0.67 (0.33)	0.64 to 0.7	0.63 (0.29)	0.53 to 0.73
C-16:1	-2.39 (0.35)	-2.42 to -2.36	-2.31 (0.36)	-2.43 to -2.19
C-16:1OH	**-3.18 (0.33)**	**-3.21 to -3.16**	**-3.35 (0.28)**	**-3.44 to -3.26**
C-18	-0.24 (0.34)	-0.26 to -0.21	-0.32 (0.3)	-0.42 to -0.22
C-18:1	-0.36 (0.33)	-0.39 to -0.33	-0.31 (0.31)	-0.42 to -0.21
C-18:2	-1.88 (0.34)	-1.91 to -1.85	-1.88 (0.27)	-1.97 to -1.79
**Amino Acids/Intermediates**				
Alanine	5.51 (0.24)	5.49 to 5.53	5.51 (0.29)	5.41 to 5.61
Arginine	2.25 (0.51)	2.21 to 2.29	2.35 (0.54)	2.17 to 2.54
Citrulline	2.32 (0.26)	2.3 to 2.34	2.23 (0.28)	2.14 to 2.33
Glutamate	5.04 (0.26)	5.02 to 5.06	5.08 (0.33)	4.97 to 5.19
Leucine	**4.7 (0.21)**	**4.68 to 4.72**	**4.83 (0.28)**	**4.73 to 4.92**
Methionine	2.99 (0.18)	2.97 to 3	3.02 (0.28)	2.93 to 3.11
Ornithine	2.95 (0.3)	2.92 to 2.97	2.86 (0.34)	2.75 to 2.98
Phenylalanine	**4.18 (0.2)**	**4.17 to 4.2**	**4.32 (0.25)**	**4.24 to 4.41**
Succinylacetone	-0.7 (0.22)	-0.72 to -0.69	-0.72 (0.25)	-0.81 to -0.64
Tyrosine	**3.79 (0.21)**	**3.78 to 3.81**	**3.9 (0.23)**	**3.82 to 3.98**
Valine	4.73 (0.17)	4.71 to 4.74	4.79 (0.2)	4.72 to 4.85
**Hormones**				
17-Hydroxyprogesterone	4.97 (1.1)	4.88 to 5.06	4.9 (1.4)	4.43 to 5.37
Thyroid Stimulating Hormone	1.78 (0.74)	1.72 to 1.84	1.75 (0.75)	1.5 to 2.01

All variables are natural log transformed

Supplemental Table S4. Cross validated multivariable logistic cord model built within the Busia cohort.

	**Cord**	
	AUC: 0.935 95% CI: 0.894-0.977	
Variable	Parameter Estimate	OR (95% CI)
Intercept	8.63	NA
Birthweight (per 100g)	-0.55	0.62 (0.54-0.73)
Alanine	-0.72	0.08 (0.01-0.57)
C4	-2.53	0.18 (0.03-0.94)
C4-DC	-0.94	16.78 (2.85-98.78)
C4-OH	-2.13	0.13 (0.03-0.56)
C16:1-OH	-1.95	3.45 (1.09-10.94)

Supplemental Table S5. Classification statistics of cord models used to determine preterm birth

	**Cord**	
	Ryckman	Busia
Sensitivity	64.1	52.8
Specificity	83.6	99.2
Positive Predictive Value	52.1	79.2
Negative Predictive Value	84.8	97.2
Accuracy	82.4	96.5

Supplemental Table S6. Weeks of difference between cord model determined gestational ages and ultrasound determined gestational ages.

	**Ryckman Cord**		**Busia Cord**	
	Frequency (%)	Cumulative Frequency (%)	Frequency (%)	Cumulative Frequency (%)
Perfect Match	23 (3.6)	23 (3.6)	37 (5.8)	37 (5.8)
≤1 Week	210 (32.8)	233 (36.4)	353 (55.2)	390 (60.9)
≤2 Weeks	203 (31.7)	436 (68.1)	181 (28.3)	571 (89.2)
≤3 Weeks	128 (20.0)	564 (88.1)	51 (8.0)	622 (97.2)
≤4 Weeks	52 (8.1)	616 (96.3)	11 (1.7)	633 (98.9)
≤5 Weeks	17 (2.7)	633 (98.9)	6 (0.9)	639 (99.8)
5+ Weeks	7 (1.1)	640 (100.0)	1 (0.2)	640 (100.0)

*Perfect Match is ±1/2 day.

Supplemental Table S7. Weeks of difference between cord model determined gestational ages and ultrasound determined gestational ages in infants born SGA.

	**Ryckman Cord**				**Busia Cord**			
	Intergrowth SGA		Busia Specific SGA		Intergrowth SGA		Busia Specific SGA	
	No SGA (n = 534)	SGA (n = 106)	No SGA (n = 556)	SGA (n = 84)	No SGA (n = 534)	SGA (n = 106)	No SGA (n = 556)	SGA (n = 84)
Perfect Match*	23 (4.3)	0 (0.0)	23 (4.1)	0 (0.0)	33 (6.2)	4 (3.8)	36 (6.5)	1 (1.2)
0-1 Week	206 (38.6)	4 (3.8)	204 (36.7)	6 (7.1)	313 (58.6)	40 (37.7)	327 (58.8)	26 (31.0)
1-2 Weeks	181 (33.9)	22 (20.8)	188 (33.8)	15 (17.9)	147 (27.5)	34 (32.1)	154 (27.7)	27 (32.1)
2-3 Weeks	96 (18.0)	32 (30.2)	110 (19.8)	18 (21.4)	29 (5.4)	22 (20.8)	28 (5.0)	23 (27.4)
3-4 Weeks	21 (3.9)	31 (29.3)	24 (4.3)	28 (33.3)	7 (1.3)	4 (3.8)	6 (1.1)	5 (6.0)
4-5 Weeks	6 (1.1)	11 (10.4)	6 (1.1)	11 (13.1)	4 (0.8)	2 (1.9)	4 (0.7)	2 (2.4)
5+ Weeks	1 (0.2)	6 (5.7)	1 (0.2)	6 (7.1)	1 (0.2)	0 (0.0)	1 (0.2)	0 (0.0)

Values are frequency (column %). Busia specific SGA determined using WHO calculator

*Perfect Match is ±1/2 day.
